# Comparative studies of *Toxoplasma gondii* transcriptomes: insights into stage conversion based on gene expression profiling and alternative splicing

**DOI:** 10.1186/s13071-018-2983-5

**Published:** 2018-07-11

**Authors:** Long-Fei Chen, Xiao-Long Han, Fen-Xiang Li, Yun-Ying Yao, Jin-Ping Fang, Xiao-Ju Liu, Xiao-Cong Li, Kun Wu, Min Liu, Xiao-Guang Chen

**Affiliations:** 1 Department of Pathogen Biology, Guangdong Provincial Key Laboratory of Tropical Disease Research, School of Public Health, Southern Medical University, Guangzhou North Avenue No.1838, Guangzhou, 510515 Guangdong China; 2 Department of Bioinformatics, School of Basic Medicine School of Basic Medicine, Southern Medical University, Guangzhou, 510515 Guangdong China; 3 Epidemiology and Infection Control Branch, Shenzhen Guangming District Center for Disease Control and Prevention, Shenzhen, 518106 Guangdong China; 4 First Clinical Medical College, Southern Medical University, Guangzhou, 510515 Guangdong China

**Keywords:** Gene expression, RNA-seq, Transcriptome, mRNAs, Alternative splicing, *Toxoplasma gondii*

## Abstract

**Background:**

*Toxoplasma gondii* is one of the most important apicomplexan parasites and infects one-third of the human population worldwide. Transformation between the tachyzoite and bradyzoite stages in the intermediate host is central to chronic infection and life-long risk. There have been some transcriptome studies on *T. gondii*; however, we are still early in our understanding of the kinds and levels of gene expression that occur during the conversion between stages.

**Results:**

We used high-throughput RNA-sequencing data to assemble transcripts using genome-based and *de novo* strategies. The expression-level analysis of 6996 *T. gondii* genes showed that over half (3986) were significantly differentially expressed during stage conversion, whereas 2205 genes were upregulated, and 1778 genes were downregulated in tachyzoites compared with bradyzoites. Several important gene families were expressed at relatively high levels. Comprehensive functional annotation and gene ontology analysis revealed that stress response-related genes are important for survival of bradyzoites in immune-competent hosts. We compared Trinity-based *de novo* and genome-based strategies, and found that the *de novo* assembly strategy compensated for the defects of the genome-based strategy by filtering out several transcripts with low expression or those unannotated on the genome. We also found some inaccuracies in the ToxoDB gene models. In addition, our analysis revealed that alternative splicing can be differentially regulated in response to life-cycle change. In depth analysis revealed a 20-nt, AG-rich sequence, alternative splicing locus from alt_acceptor motif search in tachyzoite.

**Conclusion:**

This study represents the first large-scale effort to sequence the transcriptome of bradyzoites from *T. gondii* tissue cysts. Our data provide a comparative view of the tachyzoite and bradyzoite transcriptomes to allow a more complete dissection of all the molecular regulation mechanisms during stage conversions. A better understanding of the processes regulating stage conversion may guide targeted interventions to disrupt the transmission of *T. gondii*.

**Electronic supplementary material:**

The online version of this article (10.1186/s13071-018-2983-5) contains supplementary material, which is available to authorized users.

## Background

*Toxoplasma gondii* belongs to the phylum Apicomplexa, which includes many other deadly pathogens, such as *Plasmodium*, responsible for malaria, and *Cryptosporidium*, the cause of cryptosporidiosis [1]. Because of its remarkable capacity for invasion, transmission, and persistence, it is estimated that one-third of the human population is chronically infected with *T. gondii*. The reported prevalence rates range from a few percent to nearly 80%, depending on the population [2–4]. *Toxoplasma gondii* is one of the most important opportunistic pathogens in immune-compromised individuals, including patients with acquired immune deficiency syndrome (AIDS)/human immunodeficiency virus (HIV) and those receiving cancer treatments or organ transplants [3, 5]. It can also cross the placental barrier and cause abortion or congenital birth defects [6]. *In utero* infection may elevate the risk ofocular toxoplasmosis owing to spontaneous reactivation of the disease [7].

While the complex life-cycle of *T. gondii* includes sexual and asexual stages in felines and intermediate hosts, respectively, it has an unusual capability to clonally propagate in intermediate hosts. The asexual life-cycle of *T. gondii* occurs within all intermediate hosts and involves a conversion between two distinct life forms: an initial phase with rapid multiplication of tachyzoites that are responsible for the acute stage of the disease, and a slowly dividing or resting phase with bradyzoites that are contained in thick-walled tissue cysts to evade the host immune system [8–10]. The latent bradyzoite cysts usually form in brain, skeletal muscles, or visceral organs, and are responsible for chronic disease because of their ability to evade the immune system, to resist commonly used drug treatments, and to reactivate into virulent tachyzoites [11–13]. As a consequence, it is essential to evaluate the regulation of gene expression during the critical interconversion between these two stages.

*Toxoplasma gondii* maintained in cell culture are capable of stage interconversion and have been used to explore the differences in the transcripts between tachyzoites and bradyzoites (tissue cysts) [14]. However, the percentage of cells forming cysts is low and gene expression is asynchronous. Previous studies of the parasite’s life-cycle stages have indicated a complex pattern of expression associated with each of its forms. Serial analysis of gene expression (SAGE) has demonstrated that 18% of the tags were marked by unique stage-specific mRNA during its life-cycle [15]. Some of the genes specifically regulated during the tachyzoite-bradyzoite transition are expressed throughout mitosis, cytokinesis, and early G1 phases, in concordance with the cell cycle arrest observed during bradyzoite differentiation [16]. The recent development of next-generation sequencing (NGS) technology has commenced a new era for *Toxoplasma* genomics and transcriptome studies. Several databases of *T. gondii* have been constructed on ToxoDB for use in research, representing the predominant strains in Europe and North and South America [17]. Using NGS to sequence full-length cDNAs (FL-cDNAs) reversely transcribed from RNAs, the RNA-sequence (RNA-Seq) technology provides a new approach for studying transcriptomes [18, 19]. This RNA-Seq can be used not only to measure gene expression levels at higher resolution than microarrays, but can also reveal unknown transcripts and splicing isoforms and provide a quantitative measurement of alternatively spliced (AS) isoforms. Such new possibilities are expanding the transcriptome studies of *T. gondii* from conventinal gene expression studies to all aspects of transcriptomes, including AS, gene fusion, and various kinds of non-coding RNAs [18]. However, to the best of our knowledge, there have been no reports on the comparative analysis of transcriptomes from different stages of PRU strain *T. gondii*, especially from the tissue cyst bradyzoites.

In this study, we tried to delineate the transcriptomes of bradyzoites *in vivo* and compare them with those of tachyzoites from the same strain of *T. gondii*. The genome-based assembly method was utilized for relative comparisons of gene expression in the different stages, while Trinity *de novo* assembly was used for genome annotation and AS exploration. Our findings demonstrated major changes in gene expression between tachyzoites and bradyzoites in *T. gondii*, and provided insight into the pathways regulating these processes. A better understanding of the processes regulating stage conversion may help guide targeted interventions to disrupt transmission of this deadly parasite.

## Methods

### Parasitology

The TypeII *T. gondii* Prugniaud (PRU) strain was kindly provided by the Department of Parasitology, Xinxiang Medical College, Henan, China. Tachyzoites of *T. gondii* were collected from invaded HFF cells *in vitro* and purified by a 3-μm membrane filter. Bradyzoites (tissue cysts) were harvested from infected mice brains and purified by Ficoll (Sigma Co. Ltd) density gradient centrifugation as previously described [20].

### RNA preparation and sequencing

Tachyzoites were washed three times with 1× phosphate-buffered saline (Invitrogen Co. Ltd) and collected by centrifugation at 1500× *rpm* for 5 min after induction to wash off the medium. Bradyzoites were lysed from tissue cysts by using 0.25% pancreatic enzyme (Invitrogen Co. Ltd). Total RNA was extracted from the purified tachyzoites and bradyzoites of the *T. gondii* PRU strain by using Trizol reagent according to the manufacturer’s protocol (Invitrogen Co. Ltd). The RNA concentration for each sample was measured using Nanodrop (Thermo Scientific Co. Ltd) and the Agilent 2000 Bioanalyzer (Agilent Technologies Co. Ltd).

### Illumina sequencing and filtering

The RNA samples were processed by high-throughput cDNA sequencing (RNA-Seq) using Illumina HiseqTM 2000 at the Beijing Genomics Institute (BGI) - Shenzhen, China. Each sequencing feature can yield 2 × 90 base pairs (bp) independent reads from either end of a fragment. Sequencing errors can create difficulties for the short-read assembly algorithm. Therefore, we conducted a stringent filtering process of raw sequencing reads before the transcriptome assembly. We removed the raw low-quality reads (more than 5% ambiguous sequences “N” and more than 10% bases with a quality score of Q < 20) with an adaptor. Then, the high-quality transcriptome sequence data was mapped to mouse (mm9) and human (hg18) genomes using Bowtie2 [21] (v 2.1.0) and TopHat2 [22] (v 2.0.9) with the default parameters set to filter host genes. The remaining clean reads were considered to be *T. gondii* reads and used for the downstream informatic analyses.

### Transcriptome assembly and annotation

Two libraries of *T. gondii* PRU strains were assembled with two different strategies: (i) a *de novo* assembly pipeline, utilizing the Trinity assembly suite [23]; and (ii) a genome-guided assembly pipeline, performed according to the Cufflinks protocol [24].

In the *de novo* pipelines, transcriptome assembly was performed on the clean reads using the short read assembly program Trinity (release-20130225). Trinity, consisting of three distinct software modules (Inchworm, Chrysalis, and Butterfly), had a consistently better integrated performance than most other single k-mer assemblers [25]. We needed more complete transcripts for further annotation and analysis in our research. We used the eukaryotic genome annotation tool, which is the program used to assemble spliced alignments (PASA) [26] (PASA_r20140417), to reconstruct the Trinity-assembled contigs on the *T. gondii* Me49 genome (ToxoDB, version 11.0), as performed in previous research [18]. Because of potential errors in sequencing and Trinity assembly, PASA first aligned the contigs to the Me49 genome using the Genome Mapping and Alignment Program (GMAP) [27] (version 2014-04-20); the valid contigs were then reconstructed into complete transcripts.

For the genome-guided transcriptome assembly pipeline, Bowtie2 and Tophat2 first needed to align the reads to the Me49 genome. Then, Cufflinks assembled the aligned RNA-Seq reads into transcripts by taking the binary version of the sequence alignment data as input and measuring transcript abundances in fragments per kilobase of exon per million fragments mapped (FPKM). Finally, we used the Cuffmerge program to merge the two assembled transcripts into a final transcriptome assembly.

To complete the annotation, the transcriptome assembly results in general transfer format (GTF) from the *de novo* strategy were compared with the genome-guided strategy with the annotated Me49 genes in ToxoDB using the Cuffcompare program in the Cufflinks package.

### Identification and characterization of differentially expressed genes

We performed Cuffdiff to identify differentially expressed genes, using the negative binomial (NB) distribution for differential expression analysis (fold change ≥ 2, *P* < 0.05), on the basis of the preceding results. The Bioconductor package Cummerbund was then used to produce differential expression statistics and plots. The differentially expressed genes were searched against the Me49 annotated protein database (e-value < 1e-10). For further characterization, the differentially expressed genes were also searched against the nonredundant protein (Nr) database using Blastx with an e-value cut-off of 10e-10. With Nr annotation, we used the Blast2GO program [28] to obtain gene ontology (GO) annotation of genes. The WEGO software [29] was used to perform GO functional classification to understand the distribution of gene functions.

### Structure annotation and assessing alternative splicing

For the purpose of gene structure annotation and analysis, we used BEDTools [30] (v 2.17.0) to search for overlaps between the PASA transcripts and the Me49 gene coordinates. The overlapping transcripts were searched against the annotated Me49 protein database using Blastx with an e-value cut-off of 1e-10.

PASA also identified and classified all splicing variations supported by incompatible transcript alignments. The unoverlapping transcripts were also searched against the NCBI Nr database (e-value < 1e-10). These matching transcripts obtained from the two preceding Blastx processes, re-submitted together into PASA with the parameter “--ALT SPLICE,” to identify and classify potential AS between the type II *T. gondii* strains. The MEME suite [31] provides a motif discovery algorithm, which has been widely used for DNA and protein sequence motif discovery. Because of AS, sites usually have fixed patterns on both ends, so we used MEME to assess the predicted AS sequences.

The structure and AS model were further confirmed by viewing the PASA and ToxoDB gene annotation files in the Integrative Genomics Viewer (IGV) [32].

## Results and discussion

### Genome-based assembly and analysis

We sequenced the transcriptomes of *T. gondii* tachyzoites and bradyzoites in order to understand changes in gene regulation during stage conversion in intermediate hosts. We used tachyzoites purified *via* *in vitro* culture and bradyzoites (tissue cysts) harvested from the brains of orally infected mice for RNA analysis. Illumina sequencing generated a total of 8 G 90 bp short reads from the tachyzoites and bradyzoites of the *T. gondii* PRU strain. We initially used Tophat to filter out the mouse and human reads. Using Tophat, a total of 50 and 47 M clean reads for tachyzoites and bradyzoites, respectively, were aligned to the *T. gondii* Me49 genome assembly, another type II strain. The mapping statistics showed that the proportion of the sequence that aligned with the reference genome was 87.15% and 94.1% in tachyzoites and bradyzoites, respectively, which was comparable to published data. The unmapped sequences in each library were only partially (< 13%), accounting for by tRNA or rDNA genes, which was not represented in the genome assembly.

### Gene expression changes during stage conversion

**Fig. 1:**
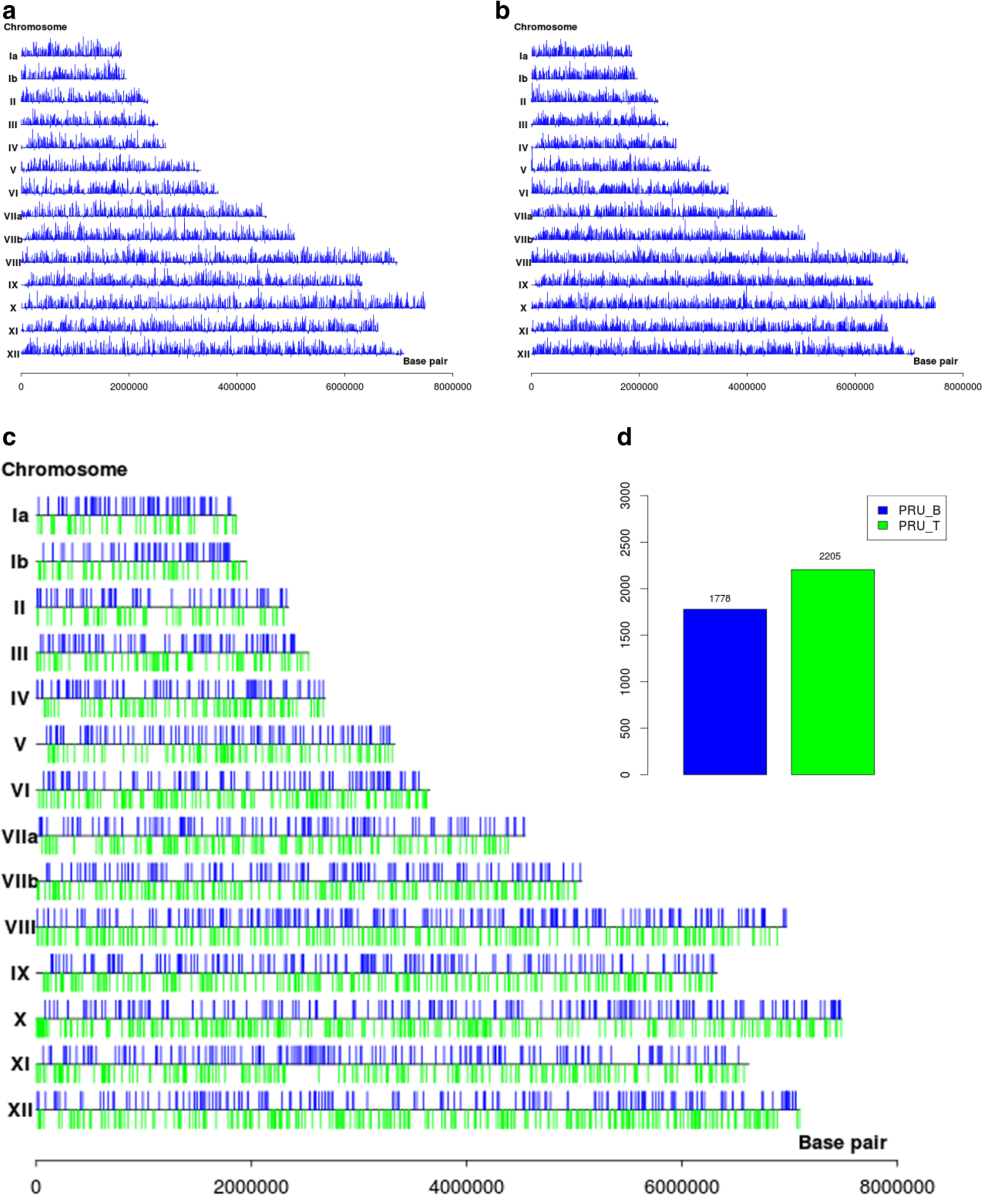
The mRNA expression across the chromosomal arm of *T. gondii*. The density of expressed genes across the chromosomal arm of tachyzoites (**a**) and bradyzoites (**b**). **c** The chromosome landscape of differentially expressed genes of *T. gondii* (fold change ≥ 2, *P* < 0.05). The differential gene expressions for *T. gondii* are indicated by the blue bar (bradyzoite upregulation) and green bar (tachyzoite upregulation). **d** The statistics of the differentially expressed genes of *T. gondii*

We examined the gene expression levels of the transcripts in tachyzoites and bradyzoites in order to explore the transcriptional changes between the two stages. The normalized expression values for all the expressed genes were recorded as FPKM using Cufflinks, which showed that the majority (6996/8155) [17] of the gene models in the genome had been covered. The tachyzoite and bradyzoite transcript libraries had different median read coverage values of 10.4 and 6.5 FPKM, respectively. Cufflinks generated a total of 15,323 unique transcripts from both libraries combined (Additional file 1: Table S1). The transcripts were distributed across the 14 *T. gondii* chromosomes (Fig. 1a, b), indicating no artificial bias according to the genome distribution. Most of the genes were expressed at both stages, with 36 specific expression genes in bradyzoites and 185 in tachyzoites. We identified significantly differentially expressed genes (fold change ≥ 2, *P* < 0.05) comparison. The data of the FPKM values and significantly differentially expressed genes are shown in Additional file 2: Table S2. We compared the expression levels using statistical pairwise comparisons of stages to identify significantly up- and downregulated genes with chromosome distribution (Fig. 1c). As previously reported [33], many genes were upregulated (2205), but fewer genes (1778) were downregulated in tachyzoites when compared with bradyzoites (Fig. 1d). The transcriptional changes between tachyzoites and bradyzoites suggested a broad shift gene activation with packaged DNA in the unicellular *T. gondii*. Overall, over half of all *T. gondii* expressed genes (3986/6996) were significantly differentially expressed during stage conversion. A similar scale of change in the transcriptome has been reported in *Entamoeba invadens* [34], *Plasmodium* [35] and *Leishmania* [36] development. However, it sharply contrasts with results found in *Giardia lamblia* [37], where an extremely limited set of genes has shown altered expression during the life-cycle. This difference might indicate differences in the extent to which gene expression at the level of transcription, or RNA stability, regulates biological processes in these protozoa.

**Fig. 2:**
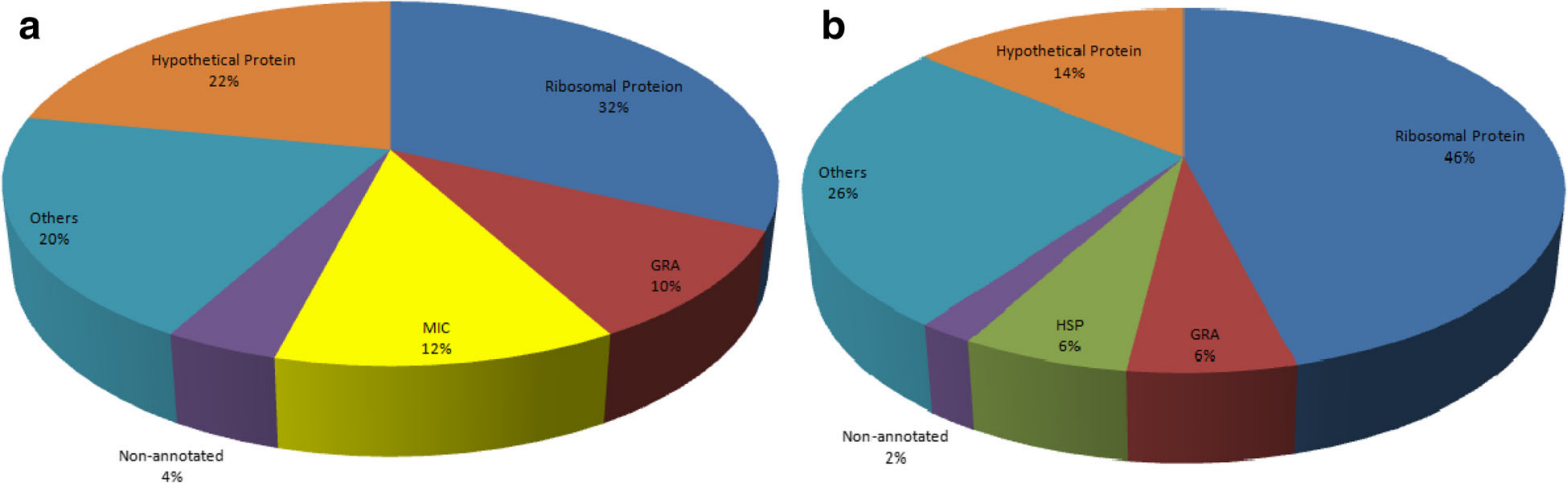
The distribution of the 50 most abundant transcripts in *T. gondii*. **a** Tachyzoites. **b** Bradyzoites. *Abbreviations*: GRA, dense granule protein; MIC, microneme protein; HSP, heat shock protein; non-annotated, non-annotated transcript on ToxoDB

Two lists with the 50 most abundant transcripts detected in each tachyzoites and bradyzoites of *T. gondii* are provided in Additional file 3: Table S3 and Additional file 4: TableS4. Several important gene families, e.g. the ribosomal protein family, dense granule protein family, microneme protein family (MIC proteins), and heat shock protein family, were expressed at a relatively high level at the top of each library (Fig. 2). The fact that the majority of the transcripts (16/50 tachyzoite and 23/50 bradyzoite) corresponded to ribosomal protein families, as occurs in another protozoan (*Leishmania* [38]), may indicate that there is generally a direct correlation between transcript levels and protein abundance in some protozoa. And the result is consistent with previous report [16] that “Ribosome Biogenesis” genes from stressed *Toxoplasma* were upregulated in comparison to unstressed one, and suggested that those ribosomal genes might be related to bradyzoite cyst formation [33, 39]. The dense granule protein (GRA protein) family was another important protein family shared by the two lists. Previous studies have shown that the secretion of the GRA proteins begins during entry of the parasite into the host cell. However, the majority occurs at the end of the invasion process, indicating that these proteins are likely involved in intracellular parasite development and multiplication [40, 41]. Another possible explanation could be that GRA expression in bradyzoites leads to accumulation of these proteins for the next infection cycle, once the parasites differentiate into tachyzoites. The abundant GRA protein family transcripts, encoding TGME49_270250 (GRA1), TGME49_227620 (GRA2), TGME49_254720 (GRA8) and TGME49_286450 (GRA5) in tachyzoites, and TGME49_288650 (GRA12) and TGME49_270250 (GRA1) in bradyzoites, were very different in kinds and levels between the two stages, apart from TGME49_203310 (GRA7). This is a surprising discovery because the GRA proteins may not only play a large role in the rapid intracellular proliferation of tachyzoites, but also function in bradyzoite (tissue cyst) formation.

At the top of the tachyzoite list was a transcript corresponding to TGME49_212200 (magnesium (Mg^2+^) transporter NIPA), a Mg^2+^ transporter distributed widely in eukaryotes with four proteins known as NIPA 1–4 [42]. It is known that organisms must maintain physiological levels of Mg^2+^, because this divalent cation is critical for the stabilization of membranes and ribosomes, for the neutralization of nucleic acids, and as a cofactor in a variety of enzymatic reactions. Bacteria have the means to assess Mg^2+^ levels in their surroundings as well as in their own cytoplasm by multiple Mg^2+^ transporters therein [43]. The ability of bacterial pathogens to detect the levels of Mg^2+^ in host tissues has shown some correlations with Mg^2+^ sensing, Mg^2+^ transport, and bacterial virulence. The ion channels in the unicellular *T. gondii* are poorly documented; thus, this finding was unexpected and there should be further exploration into how the Mg^2+^ transporters are regulated and subsequently affect the organism’s function. Other abundant transcripts included those encoding the MIC proteins, apical membrane antigen AMA1, cyclophilin, protease inhibitor (PI) 2 in tachyzoites, bradyzoite antigen (BAG1), cold/heat-shock protein family (cold-shock DNA-binding domain-containing protein, HSP90, and HSP70), elongation factor 1-alpha (EF-1-ALPHA), and facilitative glucose transporter (TgGT1) in bradyzoites. The initial discharge of the MIC proteins leads to the gliding motion necessary for invasion and attachment to the host cell [44]. According to the list, MIC10, MIC1, MIC11, MIC2-associated protein (M2AP), MIC2 and MIC6 might play a core role in this process. Research on the facilitative glucose transporter (TgGT1, TGME49_214320) in tachyzoites, which is the major hexose transporter on the parasite’s plasma membrane, has shown that it is not essential for the *in vitro* survival and *in vivo* virulence of tachyzoites [45]. The significantly increased level of TgGT1 in bradyzoites suggests that this glucose transporter might be involved in resistance to host stress in bradyzoites and formation of tissue cyst walls, similar to another nucleotide-sugar transporter (NST1) that is required for cyst wall glycosylation [46].

There were additional transcripts encoding for hypothetical proteins that were expected to be abundant. For example, CUFF.5515 would code for a hypothetical protein (of 284 amino acids), annotated as TGME49_258470, and was also identified in previous studies in other strains of *Toxoplasma*. On the other hand, three transcripts on the list (CUFF.66, CUFF.62 and CUFF.2458), which were among the most abundant transcripts, did not contain any previously annotated genes. It is clear that we urgently need more studies to understand the characteristics of these hypothetical protein genes and new transcripts that are expressed at such a high level.

### Annotation of GO terms

**Fig. 3:**
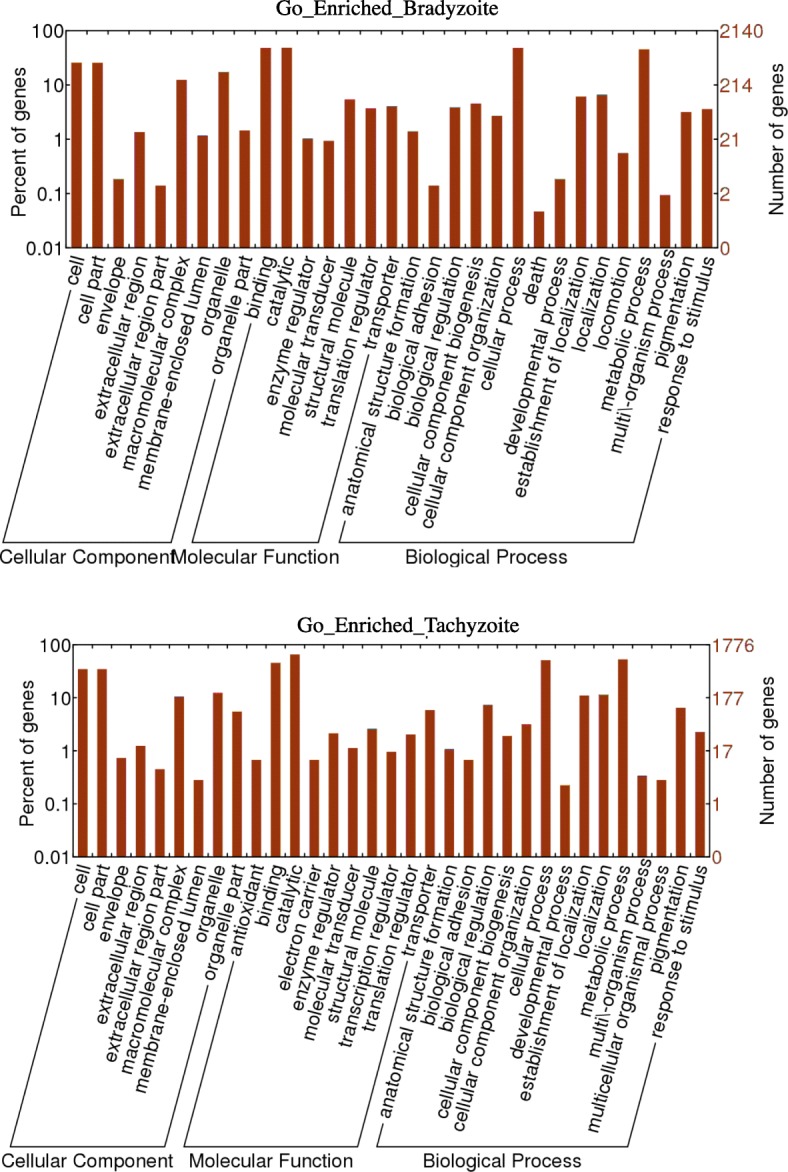
A histogram presentation of Gene Ontology (GO) classification. The bradyzoite and tachyzoite GO classification results were divided into 3 GO categories: biological processes, cellular components and molecular functions

We performed GO enrichment analysis to explore the major functional categories in the differentially expressed genes in tachyzoites and bradyzoites. The annotated GO terms were assigned to three categories, including cellular components, molecular functions, and biological processes (Fig. 3). The most common GO terms in each of the categories were “cell” and “cell part,” “binding” and “catalytic,” and “cellular process” and “metabolic process,” respectively. Compared to tachyzoite enriched cluster, the cluster “response to stimulation” and “organism process” were enriched in bradyzoite gene expression, which suggested that the bradyzoite made more effort to response to outside stimulation to help protecting the parasite.

In total, 2573 transcripts in bradyzoites and 2352 in tachyzoites were assigned to immune response-related GO classes (Additional file 5: Table S5). Transcripts related to the stress response, such as heat, cold, hypoxia, oxidative stress, and wounding, were the common stresses in GO terms. The number of stress-related transcripts in bradyzoites was two times greater than in tachyzoites. These transcripts might provide some indication of the importance of these processes to the bradyzoite’s survival in an immune-competent host.

### *De novo* full-length transcript assembly

**Fig. 4:**
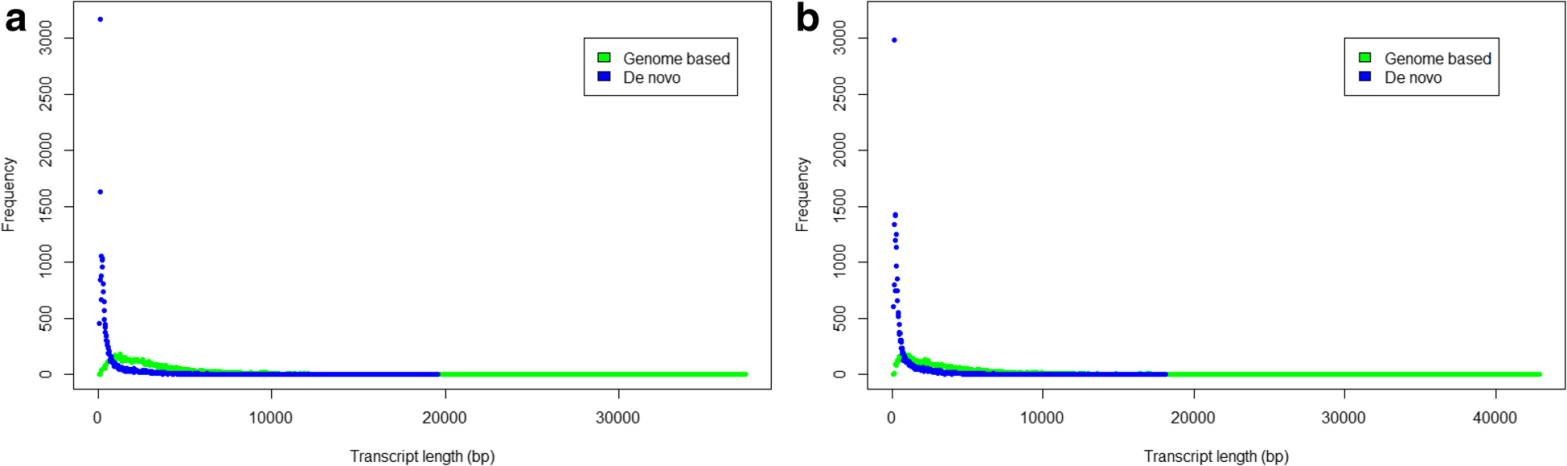
The frequency of *T. gondii* transcripts by length. The transcripts were generated by a genome-based strategy (green) and Trinity-based *de novo* strategy (blue) in bradyzoites (**a**) and tachyzoites (**b**)

A reference genome sequence is not available for many relevant parasite strains, including the *T. gondii* PRU strain. As a result, the high-throughput transcriptome analysis for characterizing candidate genes for experiments and exploiting new tools for diagnosis is limited. We conducted a Trinity-based *de novo* assembly of our reads, independent of the *T. gondii* genome sequence. The *de novo* strategy assembled 59,923 contigs in tachyzoites and 49,614 contigs in bradyzoites (Additional file 6: File S1). In summary, the *de novo* strategy generated more contigs than the genome-based assembled transcripts, but they were shorter (Table 1; Fig. 4). This strategy was able to detect 94.30% (bradyzoite) and 91.71% (tachyzoite) of the transcripts from the annotated reference genes detected by the genome-based strategy, with an additional 812 (bradyzoite) and 1514 (tachyzoite) transcripts. When we compared the transcripts predicted by both strategies, 74.97% (bradyzoite) and 72.97% (tachyzoite) of the *de novo* transcripts aligned with transcripts from the genome-based strategy, whereas 85.43% (bradyzoite) and 84.93% (tachyzoite) of the transcripts obtained with the genome-based analysis aligned with that from the *de novo* assembly. The parallel tendency of bradyzoites and tachyzoites indicated that the genome-based strategy filtered out several transcripts with low expression or were unannotated on the genome. In other words, the *de novo* assembly strategy can make up for the defects of the genome-based strategy in some cases. The *de novo* strategy does not require a reference genome for contig assembly; thus, it would still generate contigs in regions where genomic scaffolds may be missed in poorly sequenced or partially assembled genomes [47].

**Table 1:** An alignment comparison of the genome-based and *de novo* assembly strategies for the *T. gondii* transcriptome

Query	Strategy	Transcripts	AVG length (bp)	Reference genes detected	
				Shared	Unique
Bradyzoite	Genome-based	11,725	3411.79	6598	399
	*De novo*	32,769	680.71		812
Tachyzoite	Genome-based	12,393	3716.96	5603	506
	*De novo*	38,849	717.33		1514

### Gene model annotations and alternative splicing predictions

**Fig. 5:**
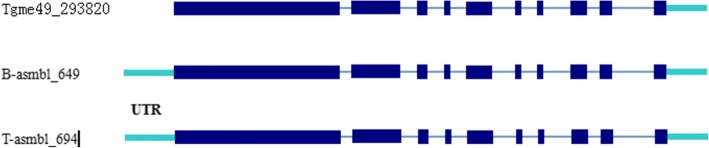
A comparison of the gene models between the currently predicted transcript and the ToxoDB transcript. A ToxoDB gene (blue) with discrepancies in the 5’ UTR compared with the PASA transcripts

Previous studies have reported that RNA-Seq is a robust and relatively inexpensive method that can be used to annotate *T. gondii* genes and transcripts by *de novo* assembly [18] (Additional file 7: file S2). Many UTR regions and exon regions were inaccurately detected in ToxoDB by IGV screening. Because of this sequencing error, we confirmed some of the UTR regions by assembled fragments that hit both of the libraries. For example, compared with TGME49_293820 (a calpain family cysteine protease domain-containing protein) in ToxoDB, the PASA transcripts in both bradyzoites and tachyzoites showed a UTR region variations in the 5′ point (Fig. 5). It is well known that the UTR regions are one of the most studied regulatory regions during biological metabolism and proliferation. The confirmed functional approach had been to combine the UTR region to degrade mRNA or prevent translation. Improving UTR annotation would be helpful to increase the understanding of the mechanisms of conversion in the *Toxoplasma* life-cycle.

**Fig. 6:**
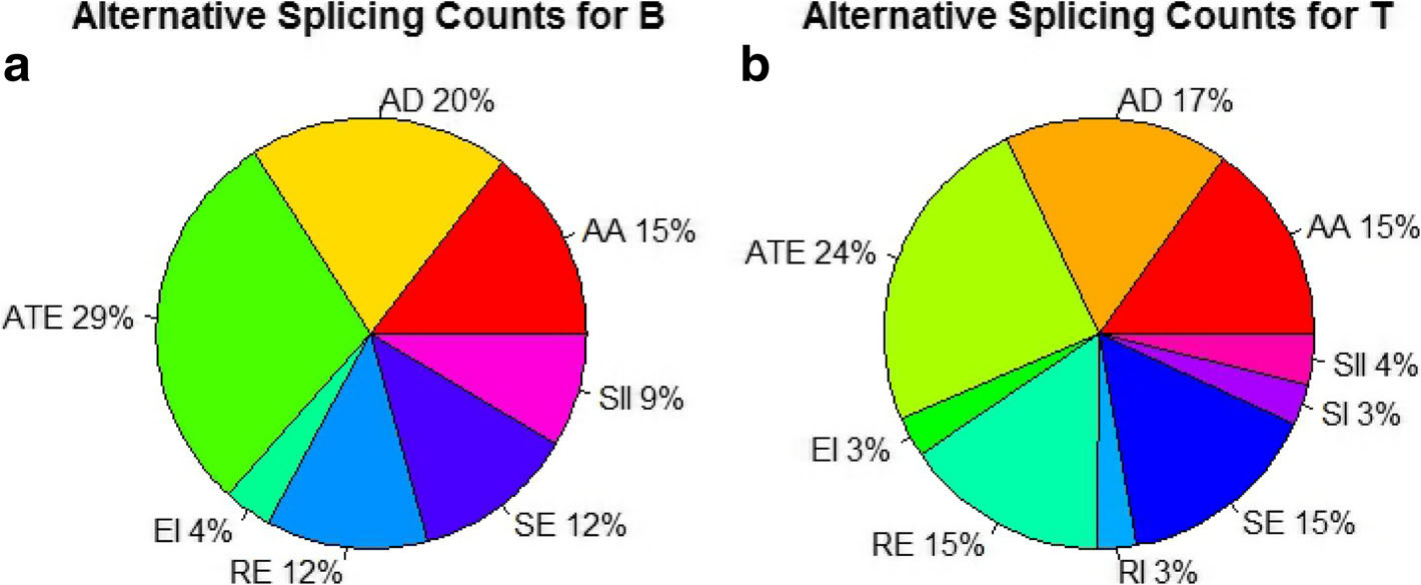
The alternative splicing classification and percentages in bradyzoites (**a**) and tachyzoites (**b**). *Abbreviations*: ATE, alternate_exon; AD, alt_donor; AA, alt_acceptor; SE, skipped_exon; RE, retained_exon; SII, starts_in_intron; SI, spliced_intron; EI, ends_in_intron

Previous large-scale studies on AS were mostly based on EST data. Although ESTs with well demarcated exon-exon junctions would be ideal for gene annotation, the high expensive cost of Sanger sequencing limits widespread use of this method. In recent years, the understanding of AS has been updated by the availability of RNA-Seq technology. We investigated the genome-wide regulation of pre-mRNA splicing in response to life-cycle changes by Trinity-based *de novo* assembled RNA-Seq because of the un-well annotated Me49 strain genome. In total, 42 genes were alternatively spliced resulting in 77 transcripts in tachyzoites, and 125 transcripts coding 65 genes were identified in bradyzoites. The AS events were classified as described in other eukaryotes (Fig. 6). Although our RNA-Seq analysis was designed for identification of AS events in tachyzoites and bradyzoites for in-depth annotation of the *T. gondii* genome, the results reflected the abundance of these events that occur during the parasite’s life-cycle. Tachyzoites and bradyzoites shared similar major types of AS events: alternate_exon (ATE), alt_acceptor (AA), alt_donor (AD), retained_exon (RE), and skipped_exon (SE) [48]. Tachyzoites expressed more alternative splicing events than did bradyzoites.

For a long time, AS was recognized as an integral part of transcriptome complexity and proteomic diversity. Alternative splicing plays a major role in the diversity and functions of proteins in cells, and aberrant splicing has been observed to be associated with many diseases [49]. We found that the AS events were obviously repressed in the dormant status (bradyzoites) and largely occurred in the rapid progenitive stage (tachyzoites), indicating some potential correlations between AS events and stage conversions.

**Fig. 7:**
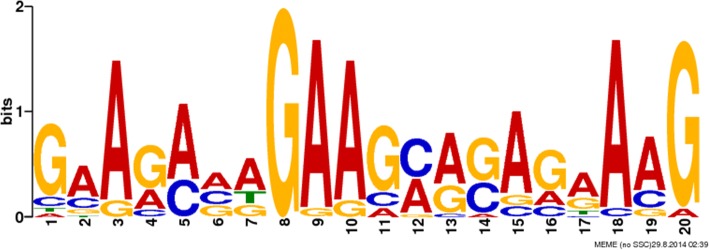
The regulatory cis-elements in the alternative splicing region of *T. gondii*. A motif search of alt_acceptor in tachyzoites yielded a 20-nt AG-rich sequence, overrepresented with an e-value of 8.2e-09

The similarities in the major types of AS events in the two stages raised another crucial question: how are AS event regulated? We made efforts to examine whether regulatory cis-elements exist in the AS regions that may control local splicing activity by using the MEME suite. A motif search of alt_acceptor in tachyzoites yielded a 20-nt AG-rich sequence, overrepresented with an e-value of 8.2e-09 (Fig. 7). The general rule is that AG-rich cis-elements are capable of promoting downstream donor site recognition in plants [50]. Such purine-rich sequences serve as the binding site of a subset of SR proteins for enhancing splicing activity in mammals [51, 52]. On the other hand, the remainder of the AS events invalided in motif searching and discovery may be regulated by other splicing cis-elements and require additional exploration.

## Conclusion

Our comparison of the tachyzoite and bradyzoite transcriptomes presented here considerably expands what is known about *T. gondii*. This study sheds light on the levels of differential gene expression, genome annotation, and AS. In addition, we found some inaccuracies in the ToxoDB gene models. A better understanding of the processes regulating stage conversion may guide targeted interventions to disrupt transmission of *T. gondii*.

## Additional files

Additional file 1: **Table S1.** The unique transcripts from tachyzoites and bradyzoites of *T. gondii*. (XLS 1379 kb)

Additional file 2: **Table S2.** The FPKM values and significantly differentially expressed genes between tachyzoites and bradyzoite of *T. gondii*. (XLS 8406 kb)

Additional file 3: **Table S3.** The lists with the 50 most abundant transcripts detected in tachyzoites of *T. gondii*. (XLS 25 kb)

Additional file 4: **Table S4.** The lists with the 50 most abundant transcripts detected in bradyzoites of *T. gondii*. (XLS 26 kb)

Additional file 5: **Table S5.** The immune response-related GO classes in tachyzoites and bradyzoites of *T. gondii*. (DOCX 19 kb)

Additional file 6: **File S1.** The *de novo* strategy assembled contigs in tachyzoites and bradyzoites of *T. gondii*. (RAR 17342 kb)

Additional file 7: **File S2.** The annotated *T. gondii* genes and transcripts by *de novo* assembly in tachyzoites and bradyzoites of *T. gondii*. (RAR 1527 kb)

## Abbreviations

AMA1: Apical membrane antigen
AS: Alternative splice
BAG1: Bradyzoite antigen
FPKM: Reads per kilobase of exon model per million mapped reads
GRA: Dense granule protein
IGV: Integrative Genomics Viewer
MIC: Microneme protein
PRU: Prugniaud
