# Supplementary material for: Comparative studies of Toxoplasma gondii transcriptomes: insights into stage conversion based on gene expression profiling and alternative splicing
Source: Parasit Vectors. 2018 Jul 11;11:402. doi: 10.1186/s13071-018-2983-5 (PMC6042387; doi:10.1186/s13071-018-2983-5)
Supplement: Supplementary file 5 — Table S5. The immune response-related GO classes in tachyzoites and bradyzoites of T. gondii. (DOCX 19 kb) [file 13071_2018_2983_MOESM5_ESM.docx]

PRU_B:

| **GO Class ID** | **Definitions** | **Counts** | **Fractions** |
| --- | --- | --- | --- |
| [GO:0008152](http://amigo.geneontology.org/cgi-bin/amigo/go.cgi?action=query&view=details&search_constraint=terms&query=GO:0008152) | metabolism | [1729](javascript:ReverseDisplay('GO:0008152')) | 67.20% |
| [GO:0019538](http://amigo.geneontology.org/cgi-bin/amigo/go.cgi?action=query&view=details&search_constraint=terms&query=GO:0019538) | protein metabolism | [453](javascript:ReverseDisplay('GO:0019538')) | 17.61% |
| [GO:0006950](http://amigo.geneontology.org/cgi-bin/amigo/go.cgi?action=query&view=details&search_constraint=terms&query=GO:0006950) | stress response | [154](javascript:ReverseDisplay('GO:0006950')) | 5.99% |
| [GO:0009056](http://amigo.geneontology.org/cgi-bin/amigo/go.cgi?action=query&view=details&search_constraint=terms&query=GO:0009056) | catabolism | [135](javascript:ReverseDisplay('GO:0009056')) | 5.25% |
| [GO:0005975](http://amigo.geneontology.org/cgi-bin/amigo/go.cgi?action=query&view=details&search_constraint=terms&query=GO:0005975) | carbohydrate metabolism | [48](javascript:ReverseDisplay('GO:0005975')) | 1.87% |
| [GO:0006629](http://amigo.geneontology.org/cgi-bin/amigo/go.cgi?action=query&view=details&search_constraint=terms&query=GO:0006629) | lipid metabolism | [30](javascript:ReverseDisplay('GO:0006629')) | 1.17% |
| [GO:0005739](http://amigo.geneontology.org/cgi-bin/amigo/go.cgi?action=query&view=details&search_constraint=terms&query=GO:0005739) | mitochondrion | [15](javascript:ReverseDisplay('GO:0005739')) | 0.58% |
| [GO:0005773](http://amigo.geneontology.org/cgi-bin/amigo/go.cgi?action=query&view=details&search_constraint=terms&query=GO:0005773) | vacuole | [5](javascript:ReverseDisplay('GO:0005773')) | 0.19% |
| [GO:0007155](http://amigo.geneontology.org/cgi-bin/amigo/go.cgi?action=query&view=details&search_constraint=terms&query=GO:0007155) | cell adhesion | [3](javascript:ReverseDisplay('GO:0007155')) | 0.12% |
| [GO:0016265](http://amigo.geneontology.org/cgi-bin/amigo/go.cgi?action=query&view=details&search_constraint=terms&query=GO:0016265) | death | [1](javascript:ReverseDisplay('GO:0016265')) | 0.04% |
| **Total** | | 2573 | 100.00% |

PRU_T:

| **GO Class ID** | **Definitions** | **Counts** | **Fractions** |
| --- | --- | --- | --- |
| [GO:0008152](http://amigo.geneontology.org/cgi-bin/amigo/go.cgi?action=query&view=details&search_constraint=terms&query=GO:0008152) | metabolism | [1466](javascript:ReverseDisplay('GO:0008152')) | 62.33% |
| [GO:0019538](http://amigo.geneontology.org/cgi-bin/amigo/go.cgi?action=query&view=details&search_constraint=terms&query=GO:0019538) | protein metabolism | [498](javascript:ReverseDisplay('GO:0019538')) | 21.17% |
| [GO:0009056](http://amigo.geneontology.org/cgi-bin/amigo/go.cgi?action=query&view=details&search_constraint=terms&query=GO:0009056) | catabolism | [149](javascript:ReverseDisplay('GO:0009056')) | 6.34% |
| [GO:0006950](http://amigo.geneontology.org/cgi-bin/amigo/go.cgi?action=query&view=details&search_constraint=terms&query=GO:0006950) | stress response | [78](javascript:ReverseDisplay('GO:0006950')) | 3.32% |
| [GO:0006629](http://amigo.geneontology.org/cgi-bin/amigo/go.cgi?action=query&view=details&search_constraint=terms&query=GO:0006629) | lipid metabolism | [65](javascript:ReverseDisplay('GO:0006629')) | 2.76% |
| [GO:0005975](http://amigo.geneontology.org/cgi-bin/amigo/go.cgi?action=query&view=details&search_constraint=terms&query=GO:0005975) | carbohydrate metabolism | [44](javascript:ReverseDisplay('GO:0005975')) | 1.87% |
| [GO:0005739](http://amigo.geneontology.org/cgi-bin/amigo/go.cgi?action=query&view=details&search_constraint=terms&query=GO:0005739) | mitochondrion | [21](javascript:ReverseDisplay('GO:0005739')) | 0.89% |
| [GO:0007155](http://amigo.geneontology.org/cgi-bin/amigo/go.cgi?action=query&view=details&search_constraint=terms&query=GO:0007155) | cell adhesion | [12](javascript:ReverseDisplay('GO:0007155')) | 0.51% |
| [GO:0000165](http://amigo.geneontology.org/cgi-bin/amigo/go.cgi?action=query&view=details&search_constraint=terms&query=GO:0000165) | MAPKKK cascade | [10](javascript:ReverseDisplay('GO:0000165')) | 0.43% |
| [GO:0043408](http://amigo.geneontology.org/cgi-bin/amigo/go.cgi?action=query&view=details&search_constraint=terms&query=GO:0043408) | regulation of MAPKKK cascade | [5](javascript:ReverseDisplay('GO:0043408')) | 0.21% |
| [GO:0009628](http://amigo.geneontology.org/cgi-bin/amigo/go.cgi?action=query&view=details&search_constraint=terms&query=GO:0009628) | response to abiotic stimulus | [1](javascript:ReverseDisplay('GO:0009628')) | 0.04% |
| [GO:0005773](http://amigo.geneontology.org/cgi-bin/amigo/go.cgi?action=query&view=details&search_constraint=terms&query=GO:0005773) | vacuole | [1](javascript:ReverseDisplay('GO:0005773')) | 0.04% |
| [GO:0006897](http://amigo.geneontology.org/cgi-bin/amigo/go.cgi?action=query&view=details&search_constraint=terms&query=GO:0006897) | endocytosis | [1](javascript:ReverseDisplay('GO:0006897')) | 0.04% |
| [GO:0006909](http://amigo.geneontology.org/cgi-bin/amigo/go.cgi?action=query&view=details&search_constraint=terms&query=GO:0006909) | phagocytosis | [1](javascript:ReverseDisplay('GO:0006909')) | 0.04% |
| **Total** | | 2352 | 100.00% |
